# Clo-miR-14: a medicinally valued spice-derived miRNA with therapeutic implications in rheumatoid arthritis

**DOI:** 10.1042/BSR20240311

**Published:** 2024-09-10

**Authors:** Ashish Sarkar, Mohd Saquib, Debolina Chakraborty, Sonia Mann, Swati Malik, Prachi Agnihotri, Lovely Joshi, Rajesh Malhotra, Sagarika Biswas

**Affiliations:** 1 Council of Scientific & Industrial Research (CSIR)-Institute of Genomics and Integrative Biology, Delhi University Campus, Mall Road, Delhi, 110007, India; 2 Academy of Scientific and Innovative Research (*AcSIR*), Ghaziabad, Uttar Pradesh 201002, India; 3 All India Institute of Medical Science (AIIMS), Ansari Nagar, New Delhi 110029, India

**Keywords:** gene expression and regulation, medicinal chemistry, microRNA, nuclear factor kappaB, rheumatoid arthritis

## Abstract

Plant microRNAs (miRNA) are regularly consumed orally along with diet, gaining attention for their RNA-based drug potential because of their ability to regulate mammalian gene expression specifically at the post-transcriptional level. Medicinally valued plants are well known for their anti-inflammatory property; however, the contribution of their miRNA in managing inflammation has been less studied. We investigated miRNA from four medicinally valued regularly consumed spices, and validated one of the most potential miRNA ‘Clo-miR-14’ for its thermal stability, and absorption in the plasma samples of RA patient’s by RT-PCR. *In vitro* and *in vivo* studies were performed to investigate the effect of Clo-miR-14 in ameliorating rheumatoid arthritis (RA) like symptoms. Our results suggest that ‘Clo-miR-14,’ an exogenous miRNA present in Curcuma longa, absorbed through regular diet, has robust thermal stability at 100°C in humans. It significantly reduced pro-inflammatory cytokines (TNF, IL-1β, IL-6) and RA-like symptoms, suggesting that plant-based miRNA could be a promising candidate as an RNA-based drug for RA pathogenesis.

## Introduction

Micro RNAs (miRNAs) are small (19–24 nucleotide) non-coding nucleotide RNAs from the hairpin precursor that regulate gene expression by pairing with 3´ untranslated regions of target genes [1]. Evidence of horizontal transfer of miRNAs between kingdoms has recently been demonstrated, suggesting a novel role for these molecules in inter-kingdom communication [2]. The circulating miRNAs are transported naturally via microvesicles that specifically get released during circulation at the target organ/cells by various routes, resulting in the regulation of the target gene [3]. The first evidence of miRNAs was demonstrated in rice plants by L. Zhang, 2012 [4], regulates human LDLRAP1 gene by MIR168a. Afterwards, various miRNAs were identified targeting specific genes in humans [2,5]. The dietary intake of miRNAs, their absorption, and circulation in the blood raised various questions about their stability in the biological fluids such as in blood, milk, as well as in extreme pH conditions during the digestion process [4,5]. The regulatory role of miRNAs is well established in humans and in many diseases such as in rheumatoid arthritis (RA), where miR-222, miR-532, miR-98, and miR-92a were found to be differentially regulated and also linked to the gender biasness of the disease [6]. Various *in silico* and *in* *vitro* studies reported to show that specific miRNA from edible/medicinal valued plants can specifically target mammalian genes and may help in the management of the disease symptoms [4,7].

The active compounds of medicinal-valued plants are reported to reduce symptoms of various diseases such as diabetes, atherosclerosis, allergies, and arthritis [8]. The plant parts like the rhizome of *Curcuma longa* (turmeric), seeds of *Trigonella foenum-graecum* (fenugreek), *Nigella satvia* (kalonji), and *Linnum usitatissimum* (flax) are traditionally used as medicine from decades, because of their well-known anti-inflammatory property, due to the presence of various flavonoids and active compounds [9–12]. Various studies showed that pro-inflammatory cytokines such as tumour necrosis factor-alpha (TNF-α), interleukin-1-beta (IL-1β), and IL-6 levels are actively regulated by the active compounds that are present in these medicinal valued plants [13] but, little is known about their miRNA ability to respond as an anti-inflammatory agent. Detection of miRNAs in the majority of medicinal plant extracts suggests that consuming such extracts may facilitate absorption of miRNAs, along with active compounds, contributing to the regulation of gene expression to prevent the progression of disease [5]. We, therefore randomly screened turmeric, seeds of fenugreek, kalonji, flax seed and checked potential of their miRNAs for anti-inflammatory/anti-arthritis properties. Amongst, *Curcuma longa* was screened for further study because *Curcuma longa* was predicted as a potential source of Clo-miR-14, an exogenous miRNA, showing anti-inflammatory and anti-rheumatic properties in humans in our earlier study [2]. *Curcuma longa* (turmeric) belongs to the zingiberaceae family of the plant kingdom, is considered a highly valued medicinal plant showing antioxidant, anti-inflammatory, anti-viral, antibacterial, antifungal, and anticancer properties and is a part of regular diet [14].

Although, previously we reported that Clo-miR-14 miRNA in *Curcuma longa* exhibited a potential anti-arthritis property, shown stable in fetal bovine serum (FBS) and is broadly consumed along with cooked food [2,15], but its thermal stability remained unanswered. We, therefore, focused our research work on validating, and quantitating the presence of Clo-miR-14 in the human plasma samples, checked its thermal stability at 100°C followed by *in vitro* studies using SW982 cells, rheumatoid arthritis fibroblast-like synovial cells (RAFLS). Further, we conducted *in vivo* studies using collagen-induced arthritic (CIA) rat models, and validated its therapeutic potential.

## Material and methods

### Sample collection

Whole blood samples were collected in sterile ethylene diamine tetraacetic acid (EDTA) coated vial (P-Tech) from RA (*N*=8, Age 50 ± 5) and Healthy Control (HC, *N*=8, Age 50 ± 5). The biopsy synovium was also collected from RA (*N*=8) patients in Dulbecco’s Modified Eagle Medium (DMEM) supplemented with 10% Fetal Bovine Serum (FBS). All the patients included in the study fulfilled the American College of Rheumatology (ACR) criteria for the diagnosis of RA and all participating healthy volunteer has no prior history of inflammatory or joint-related disease. Patients having other comorbidity along with RA were excluded. All the volunteers included in the study have signed written consent. The study protocol is complied with the Declaration of Helsinki, approved by the ethical committee of Council of Scientific & Industrial Research-Institute of Genomics & Integrative Biology (CSIR-IGIB), New Delhi, India, and All India Institute of Medical Science (AIIMS), New Delhi, India (CSIR-IGIB/IHEC/2017-18 Dt. 08.02.2018).

### Sample preparation

The EDTA vial containing whole blood was centrifuged at 1500 × ***g*** for 10 min to separate blood plasma. The plasma was aspirated carefully and aliquoted in a centrifuge tube and stored in a −80°C deep freezer till further use [16].

### Isolation of total miRNA from plant

The Dried rhizome of *Curcuma longa* and dried seeds from the *Trigonella foenum-graecum*, *Nigella satvia*, and *Linnum usitatissimum* were washed, grind (1 g) to powder, dissolved in nuclease-free water (4 ml) separately and centrifuged at 5000 × ***g*** for 10 min. Total miRNAs were isolated from the clear supernatant using miRNeasy (Qiagen, U.S.A.) isolation kit. Briefly, 200 µl of clear supernatant was mixed with 1 ml of QIAzol lysis reagent and incubated at room temperature (RT) for 5 min; 200 µl of chloroform was added and incubated for 3 min at RT, and centrifuged at 12000 × ***g*** for 15 min. The clear top layer was mixed with ethanol (1 ml) and passed through the column (provided), washed as recommended, and miRNA was collected using 20 µl of nuclease-free water.

### Isolation of total miRNA from blood plasma

Total miRNAs were isolated from plasma samples using miRNeasy Serum/Plasma (Qiagen, U.S.A.) isolation kit [17]. Briefly, 200 µl plasma was mixed with 1 ml of QIAzol lysis reagent, incubated for 5 min at RT, and 200 µl of chloroform was added, followed by centrifugation at 12000 × ***g*** for 15 min. Supernatant was collected, mixed with 1 ml ethanol, passed through the column (provided), washed, dried, and eluted using 20 µl nuclease-free water. The concentration of total miRNAs was then measured using Nano-drop (Thermo Scientific, U.S.A.) [18].

### Total miRNA transfection and TNF-α induction

Synovial sarcoma cell line (SW982) is widely used to mimic the RA like inflammation in in-vitro model using TNF-α [18]. For total miRNA, cells (SW982) were grown in a 5% CO_2_ incubator, supplemented with complete media (DMEM with 10% FBS and 1% antibiotic solution), till 60% confluency. Transfection is the most widely used technique to deliver miRNA effectively in *in vitro* conditions, cells were transfected with total miRNA (10 ng/ml) of turmeric, fenugreek, kalonji, and flax seed using RNAiMax transfection reagents (Thermo, U.S.A.) for 48 h, which shows 60–70% transfection efficiency. The TNF-α dose has been standardized for different time point using 20 ng/ml of recombinant TNF-α using SW982 cell line. Cells were then stimulated with 20 ng/ml TNF-α for 3 h to induce inflammation [19], since TNF-α is a widely used cytokine to induce RA-like inflammation in the *in vitro* model [18].

### RNA isolation and complementary-DNA synthesis

Cells (SW982) were lysed in 500 µl TRIzol (G-bioscience) incubated for 5 min, added chloroform (200 µl), and centrifuged for 15 min at 11000 ×*** g***. The clear supernatant was mixed with isopropanol, incubated (10 min) and centrifuged for 15 min at 11000 × ***g***. RNA pellet was then washed, dissolved in 20µl nuclease-free water and concentration was measured by Nano-drop (Thermo Scientitic, U.S.A.). The first-strand complementary-DNA (c-DNA) was synthesized using c-DNA synthesis kit (G Bioscience). Briefly, 1 µg of total RNA was mixed with 1 µl Oligo dT primer and incubated at 60°C for 10 min. The reverse transcriptase mixture, along with dNTPs and RNAs inhibitor was added and incubated for 1 h at 42°C as per the manufacturer’s guidelines. The reaction was terminated by incubating the reaction mixture at 95°C for 5 min. The template cDNA was used for amplification of TNF-α, IL-1β, IL-6, and glyceraldehyde 3-phosphate dehydrogenase (GAPDH) using their respective primers and evagreen RT-qPCR master mix reagent (G Bioscience, U.S.A.). GAPDH was used as a loading control. The polymerase chain reaction (PCR) was run for 40 cycles using a light cycler-480 system (Roche, U.S.A.) and analyzed based on Δct methods [18]. The primers sequences were as follows: IL-6 (5′-GGTACATCCTCGACGGCATCT-3′, 5′-GTGCCTCTTTGCTGCTTTC AC-3′), TNF-α (5′-CCCCAGGGACCTCTCTCTAATC-3′, 5′-GGTTTGCTACAACATGGG CTACA-3′), IL-1β (5′GACCTCTGCCCTCTGGATG, 5′AGGTGCTCAGGTCATTCTCC), GAPDH (5′-GAAGGTGAAGGTCGGAGTC-3′, 5′-GAAGATGGTGATGGGA TTTC-3′).

### Polyacrylamide gel electrophoresis and SYBR staining

For denaturing urea-polyacrylamide gel electrophoresis (PAGE) analysis, 16% gel was prepared in Tris/Borate/EDTA (Tris/Borate/EDTA (TBE), 89 mM Tris-Base, boric acid, 2 mM EDTA (pH 8) buffer containing 7.5 M urea. Gel was run by applying 220 V [20], stained with SYBR gold nucleic acid stain (Invitrogen, U.S.A.) and visualized using ChemiDoc MP (Bio-Rad, U.S.A.) with Image lab software [16].

### Heat treatment of microRNA

Isolated total miRNA from *Curcuma longa* (200ng) was diluted in nuclease-free water. To check the integrity, heat treatment was given at 100°C at six different time points (20, 30, 40, 50, and 60 min), followed by cDNA synthesis (QUIGEN kit, U.S.A.) using a stem-loop primer as per the manufacturer’s guidelines [21].

### Stem-loop primer and amplification of Clo-miR-14

Stem-loop primers provide a specific and sensitive way to measure miRNA in biological samples using the reverse-transcription PCR (RT-PCR) method [22]. To measure Clo-miR-14, the total miRNA of *Curcuma longa* (200 ng) was mixed with 10 nM stem-loop primer (1µl), incubated at 60°C for 10 min for cDNA synthesis as per manufacturers guideline (QUIGEN, U.S.A.). The template cDNA was used to measure Clo-miR-14. The PCR product was run on 1.5% agarose gel, stained with EtBr and the image was taken by ChemiDoc MP (Bio-Rad, U.S.A.). The sequence of Stem-loop primer and Clo-miR-14 were as follows: Stem-loop primer for Clo-miR-14 (5-GTCGTATCCAGTGCAGGGTCCGAGGTATTCGCACTGGATACGACGCCTGGG-3) [22], Clo-miR-14 (5′-CGGCGGCTACTGTGAGTTTCC-3′, 5-GTCGTATCCAGTGCAGG GTCC-3′).

### Development of primary synovial cells from biopsy synovium

Biopsy synovium of RA patients was washed with phosphate buffer saline (PBS), adipose tissues were separated, and synovial tissues were chopped (20 g), treated with collagenase (0.5 mg/g of tissue), and incubated for 12–18 h in 30 ml complete DMEM. The tissue suspension was passed through the cell strainer (100 µ pore size, BD), cultured in a T-75 tissue culture flask in complete DMEM and used for experiments between third to fifth passages [16].

### Clo-miR-14 mimic construction and cell culture treatment

Clo-miR-14 which bears 2′-O-methylation was constructed, and transfected in the cells to find its effect on inflammation. For construction, the Clo-miR-14 miRNA sequence was taken from the literature [2]. The nucleotides were synthesized with modification at 2′-O-methylation to mimic the plant miRNA [23]. SW982 and RA primary cells (RAFLS) were grown in a 6-well plate (Nunc, U.S.A.) in complete DMEM and various assay groups (*n*=4) were made; G1-untreated Control (UT), G2- treated with transfection reagent, G3-Negative control (NC) + recombinant TNF-α, G4- 10 nM Clo-miR-14 mimic with transfection reagent+ recombinant TNF-α. Cells were transfected with Clo-miR-14 (10 nM) mimic miRNA with lipofectamine RNAi Max (Thermo, U.S.A.), incubated for 48 h, followed by TNF-α (20 ng/ml) induction in serum-free DMEM for 3 h [24].

### Western-blot

Cells (SW982 and RAFLS) were grown till 60% confluency and transfected with Clo-miR-14 as mentioned above, washed, lysed with radio-immunoprecipitation assay lysis buffer (RIPA) (Thermo, U.S.A.) containing 1% (v/v) protease and phosphatase inhibitor cocktail (Gbioscience, U.S.A.), incubated (1 h) at 4°C and centrifuged at 15000 × ***g***, at 4°C for 30 min. The supernatant was separated, and protein (40 µg) estimated by bicinchoninic acid assay (BCA) was run in Sodium dodecyl sulfate-polyacrylamide gel electrophoresis (SDS-PAGE) gel (12%) and transferred on nitrocellulose (NC) membrane by semidry transfer unit (Bio-Rad, U.S.A.). The blot was then incubated overnight with 5% BSA at 4°C followed by 2 h incubation at RT with anti p-65 primary antibody (Santacruz, U.S.A.) and loading control GAPDH with 1:5000 dilution each. The blot was washed with PBS after each step, again incubated with horseradish peroxidase (HRP) conjugated anti-mouse secondary antibody (1:8000) for 45 min and the bands were observed using an enhanced chemiluminescence reagent (Cyanogen, U.S.A.). The images were obtained by Chemidoc (Bio-Rad, U.S.A.) and analyzed by image lab software (Bio-Rad, U.S.A.) [25].

### Total reactive oxygen species (ROS) estimation

SW982 cells were grown in complete DMEM, and various assay groups (*n*=4) were made; G1-UT, G2- treated with transfection reagent, G3-Negative control (NC) + recombinant TNF-α (20 ng/ml for 3 h), G4- 10 nM Clo-miR-14 mimic with transfection reagent+ recombinant TNF-α. After the treatment, cells were incubated for 30 min with a 10 μM working solution of 2′,7′-dichlorofluorescin diacetate (DCFDA) prepared in phenol red-free DMEM. Cells were then washed with PBS, followed by Fluorescence imaging by ZOE Fluorescent Cell Imager. The fluorescence intensity was analysed by ImageJ software and normalized with cell count [26].

### Development of collagen-induced arthritis (CIA) rat model

Female Wistar rats has been shown more prone to the development of RA like symptom in *in vivo* model [27]. Female Wistar rats of 4 to 6 weeks (60–80 g) were therefore procured from Indian Council of Medical Research (ICMR)-National Institute of Nutrition, Hyderabad, India. The animals were maintained, and experiments were performed at the Institute’s in-house animal facility with a controlled environment (25 ± 2°C) fed with a standard rodent chow diet and water *ad libitum*. The animals were grouped and acclimatised for two weeks. The experimental design was approved by the Institute’s Animal Ethical Committee (lGIB/IAEC/3/3/Mar 2023). Total 5 animal groups consisting of 6 animals per group were made: (1) untreated group/healthy control (HC), (2) Negative Control (NC) treated with a non-targeted miRNA pool, (3) CIA, (4) Clo-miR-14 treated group, and (5) standard drug (Methoxtrate) group. The collagen (Type II) from chicken (Sigma, U.S.A.) was thoroughly dissolved (2 mg/ml) in 0.01M acetic acid and mixed (1:1) with complete adjuvant (Sigma, U.S.A.) for induction of the CIA rats group. All groups except HC were given a collagen dose of 1 µg/g of the body weight of the rat and also miRNA 1ng/gm of the body weight of the rat [28]. The collagen dose was given subcutaneously on the first and fourteenth day through the tail vein and miRNA (Clo-miR-14) treatment was given intraperitoneally (IP) 2 days prior to the induction of CIA and thereafter on every third day till the thirteeth day. Rats were euthanized on the 34th day using Thiopentone and Xylazine (3:1) cocktail, killed, and collected blood and synovium samples to perform further assays [27].

### Assessment of macroscopic arthritis score in collagen-induced arthritis (CIA)

The arthritic score was calculated on the 28th day by macroscopic observation of swelling, edema and redness of all four paws of each rat [29]. The scores were given on a scale of 1-4: severity; 1, for no visible edema, swelling and redness; 2, for moderately involved joints; 3, for highly involved joints with edema, redness and swelling; 4, for joints severely affected by edema, swelling and redness. The swelling of joints was calculated by measuring the changes in paw volume using a plethysmometer [27].

### Detection of Clo-miR-14 level in rat synovium

The delivery of Clo-miR-14 in animals was validated in the Clo-miR-14 treated group by RT-PCR. Synovium (10 mg) samples were finely chopped, homogenized, and solubilized in TRIZOL. Total miRNA was isolated, and cDNA was prepared using stem-loop primer followed by Clo-miR-14 detection by PCR [28].

### Enzyme-linked immunosorbent assay (ELISA)

ELISA is the most sensitive and specific test to analyse biological samples. It was conducted using ELISA kits (ELK Biotechnology, China) to measure the expression level of cytokines (TNF-α and IL-6) in rat plasma [27]. Briefly, 100 µl of plasma samples were added to the pre-coated ELISA plate along with the standard (provided), incubated for 120 min at 37°C, and washed with wash buffer (provided). Biotinylated primary antibody was added to each well and incubated for 90 min at 37°C, washed after incubation, added HRP conjugated secondary antibody, and incubated for 40 min. The detection was carried out using chromophore substrate solution (provided) after washing and absorbance was taken at 462 nm by using a spectrometer (Molecular devices) [24].

### Hematoxylin and eosin staining

Rat synovium samples were fixed in 10% formalin, dehydrated, and embedded into the paraffin blocks. Blocks were cut into 5 µm thickness using microtome, sections were mounted on the slide, de-paraffinized, and stained with hematoxylin for 1 min. Slides were decolourized, counter-stained with alcoholic-eosin for 30 s, mounted with xylene on the slide, covered with a coverslip and viewed using Nikon ECLIPSE 90i microscope (NIKON, JAPAN) [30]. Images were analyzed by ImageJ (version 1.53t) software [31].

### Statistical analysis

All the statistical analysis were carried out using GraphPad Prism software (version 8.4.3). Student’s *t*-test were applied to compare variables between two groups, and one-way ANOVA was applied to compare variables among multiple groups. All the bar graphs were represented as mean ± Standard Deviation (SD) and data were represented from at least three independent experiments.

## Results

### Regulation of pro-inflammatory cytokines by medicinally valued plant miRNA

**Figure 1:**
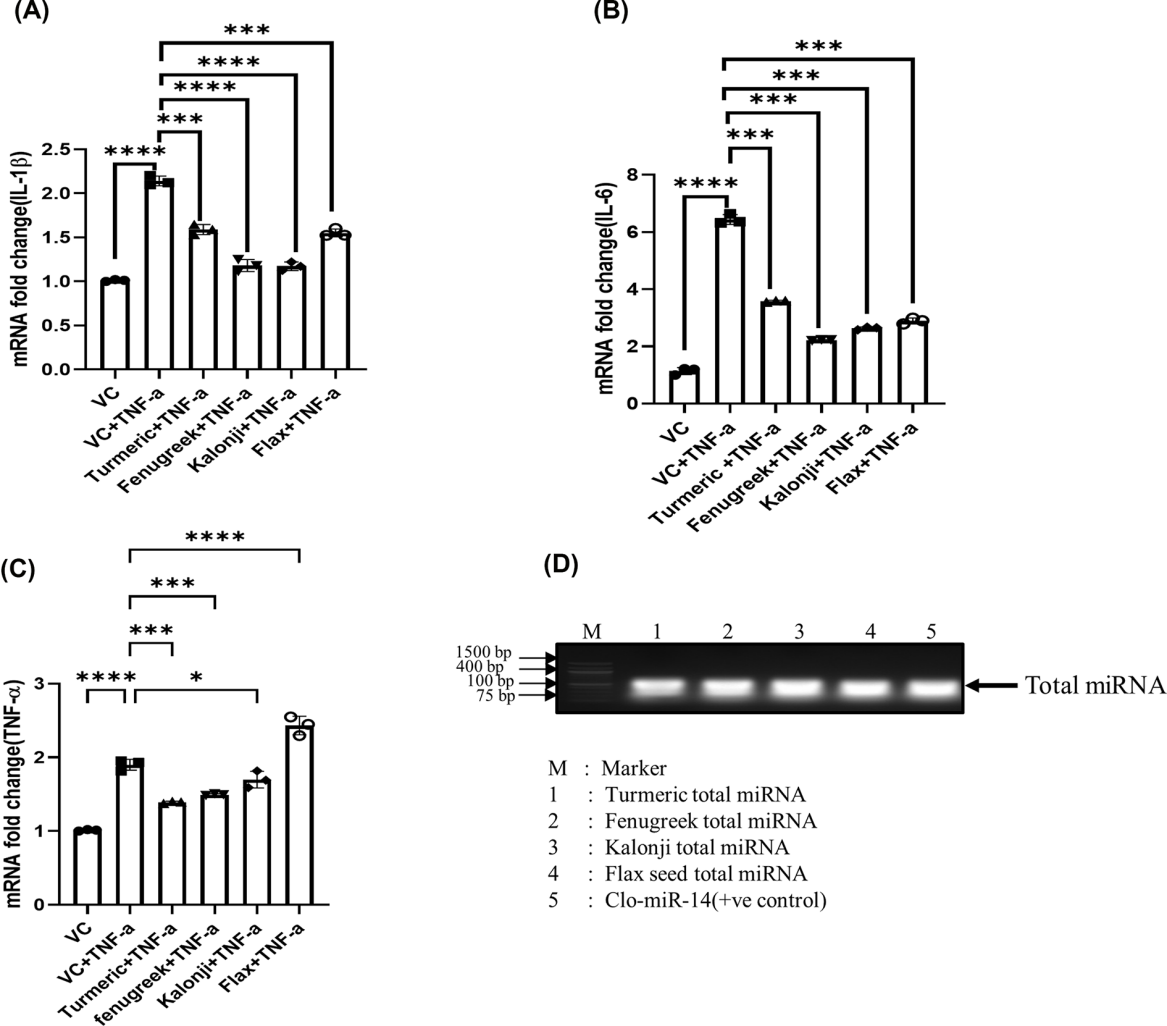
The anti-inflammatory effect of total miRNA from a medicinal plant has been checked in the SW982 cell line after inflammation induced by TNF induction (**A**) The graph represents the level of IL-1β mRNA fold change, measured after transfection with total miRNA from turmeric, fenugreek, kalonji, and flax seed. (**B**) The graph represents the level of IL-6 mRNA fold change, measured after transfection with total miRNA from turmeric, fenugreek, kalonji, and flax seed. (**C**) The graph represents the level of TNF-α mRNA fold change, measured after transfection with total miRNA from turmeric, fenugreek, kalonji, and flax seed. (**D**) The image showing agarose gel image of total microRNA from turmeric, fenugreek, kalonji, flax seed, and Clo-miR-14. The data were represented from three independent experiments and represented by a bar graph. IL, interleukin; TNF-α, tumor necrosis factor-α; VC, vehicle control; ****P≤0.0001, ***P≤0.001, **P≤0.01, *P≤0.05.

The recombinant TNF-α dose has been standardized to induce inflammation using SW982 cells (Supplementary Figure S1). Four plant spices, turmeric, fenugreek, kalonji and flax seed, miRNAs were transfected followed by TNF induction in SW982 cells, showed significantly down-regulated pro-inflammatory cytokine level: IL-1β; by 0.75- (*P*=0.0002), 0.56- (*P*=0.0001), 0.53- (*P*=0.0001), and 0.72- (*P*=0.0001) fold, respectively, compared with TNF-α treated group (Figure 1A) when cells were treated with isolated plant miRNAs. Likewise, the down-regulation of IL-6 mRNA level was found to be in turmeric, fenugreek, kalonji, and flax seed by 0.55- (*P*=0.0001), 0.34- (*P*=0.0001), 0.40- (*P*=0.0001), and 0.44- (*P*=0.0001) folds compared with TNF-α treated group (Figure 1B). We observed that the mRNA level of TNF was reduced significantly only by turmeric and fenugreek miRNAs; 0.741- (*P* =0.001) fold and 0.795- (*P*=0.0009) fold, respectively (Figure 1C) while kalonji shows non-significant changes (*P*=0.56) and flax seed shows 1.29-fold up-regulation (*P*=0.0001) compared with TNF-α treated group. The total miRNA isolated from four different medicinal valued plant also shown on agarose gel along with clo-mir-14 as positive control (Figure 1D).

Since TNF is the major pro-inflammatory cytokines involve in RA, significant down-regulation of IL-6 and IL-1β levels and non-significant reduction of TNF-α levels of kalonji and flax seed suggests that the miRNAs of kalonji and flax seed have strong anti-inflammatory effects but lack anti-rheumatic properties. Further, amongst four medicinal plant, only turmeric and fenugreek were revealed to have both anti-inflammatory effects and anti-rheumatic properties. However, turmeric was observed to show more significant anti-inflammatory and anti-rheumatic properties compared with fenugreek (Figure 1C). Turmeric was therefore selected for further study.

### Validation of Clo-miR-14 miRNA in human Plasma and *Curcuma longa*

**Figure 2:**
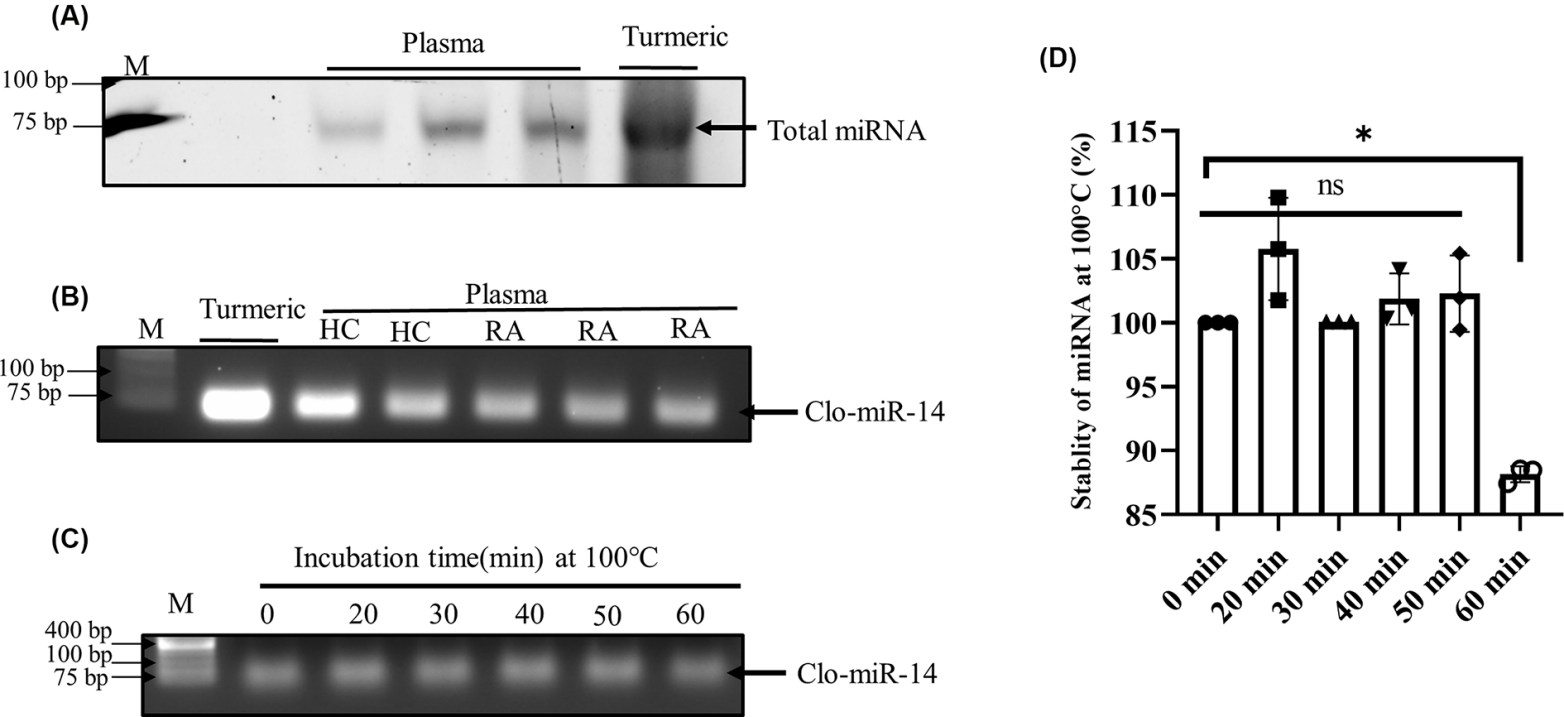
Identification and stability of miRNA isolated from *Curcuma longa* (turmeric) (**A**) Band showing total miRNA in denatured RNA gel isolated from human blood plasma (*N*=6) and curcuma longa (turmeric). (**B**) Band showing Clo-miR-14 PCR product run on 1.5% agarose gel from HC (*N*=8), RA (*N*=8), and Curcuma longa (turmeric). (**C**) Band showing Clo-miR-14 PCR product run on 1.5% agarose gel after incubation of miRNA at 100°C for 0 to 60 min. (**D**) Graph showing the level of miRNA degradation from 0 to 60 min while incubating at 100°C (cooking temperature). The graph shows three experimental replicates. M, molecular weight marker; min, minutes; *P≤0.05; HC, healthy control; PCR, polymerase chain reaction; RA, rheumatoid arthritis.

To prove that Clo-miR-14 was consumed by diet and circulated in blood, total miRNA from the human plasma samples and rhizome of turmeric were isolated and visualized in denatured RNA gel (Figure 2A). The results showed a more prominent band in the case of turmeric than plasma samples, indicating turmeric as the prominent source of Clo-miR-14. The presence of Clo-miR-14 miRNA in human blood and turmeric was then confirmed by performing PCR reaction and successively running the amplified PCR products in 1% agarose gel (Figure 2B), showing band expression of Clo-miR-14 in RA as well as in healthy plasma. However, the expression of Clo-miR-14 in turmeric (Figure 2B) was observed to be higher, indicating its abundance in the rhizome of turmeric. Further, the stability of Clo-miR-14 at cooking temperature was checked by incubating clo-mir-14 at several time points (0, 20, 30, 40, 50, and 60 min) at 100°C (Figure 2C), running PCR product on 1% agarose gel, and by analyzing PCR product (Figure 2D). Results revealed that Clo-miR-14 is stable for at least 50 min without significant changes and retained 87% integrity at 100°C (Figure 2D).

### Quantification of Clo-miR-14 in disease and healthy human plasma

**Figure 3:**
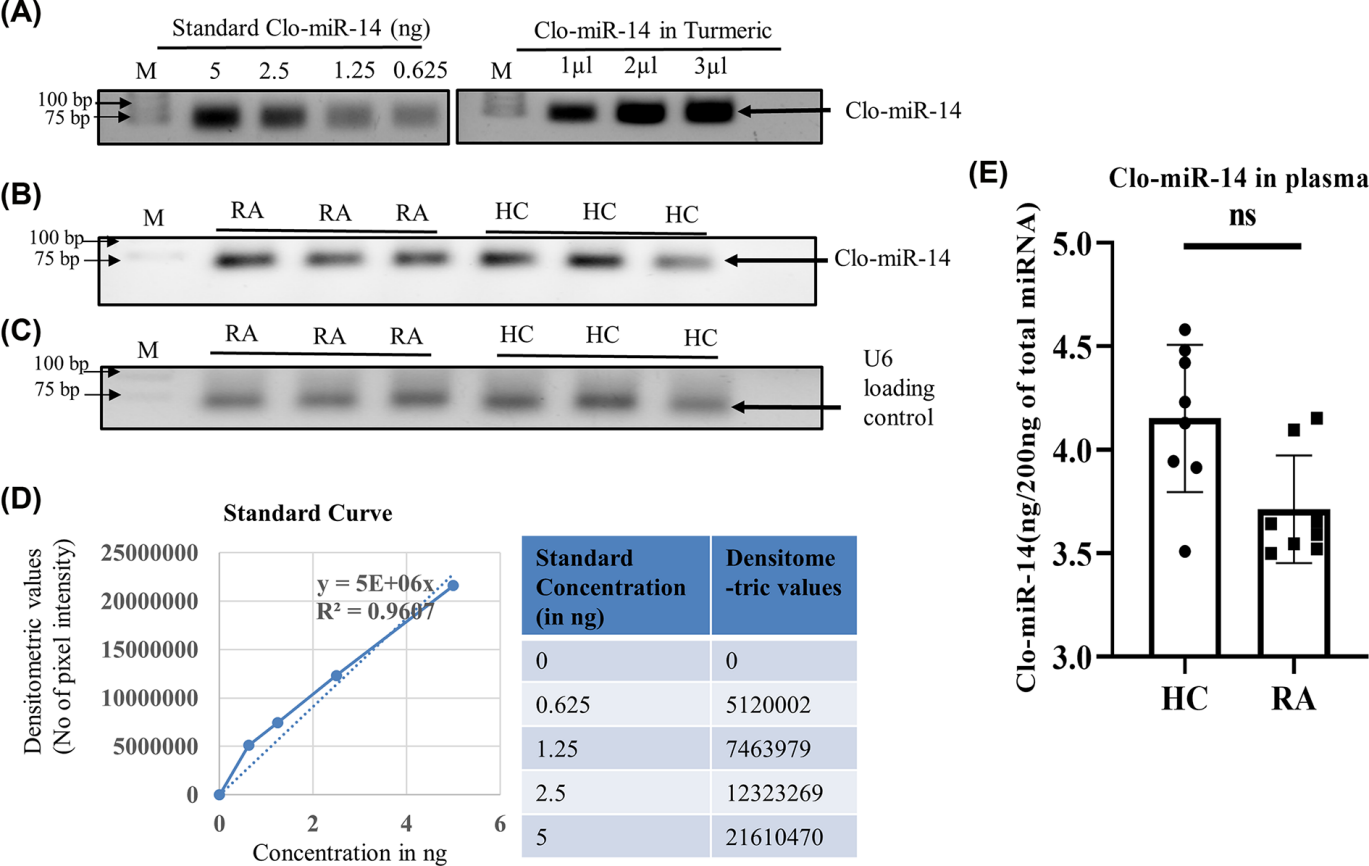
Quantification and validation of miRNA in human plasma (**A**) The image shows bands of Clo-miR-14 PCR product of commercially synthesized Clo-miR-14 using different concentrations used as a standard along with turmeric. (**B**) The band showing expression of Clo-miR-14 used to measure the concentration of Clo-mir-14 in RA (*N*=8), and HC (*N*=8) plasma samples (**C**) Band showing U6 loading control in RA (*N*=8) and HC (*N*=8) plasma samples run on 1.5% agarose gel. (**D**) The graph shows the standard curve and the table showing the values to generate the standard curve between synthetic Clo-miR-14 concentration and their corresponding densitometry values. Using the slope of the standard curve, Clo-miR-14 concentration was measured in blood plasma and the rhizome of turmeric. (**E**) The bar graph represents the concentration of Clo-miR-14 in human plasma samples, indicating a non-significant difference in RA (*N*=8) plasma samples compared with HC (*N*=8). HC, healthy control; ns, non-significant *P*-value; M, molecular weight marker; RA, rheumatoid arthritis.

Clo-miR-14 was quantified in the plasma samples of RA, HC, and turmeric rhizome. For quantification, cDNA of commercially available Clo-miR-14 was synthesized and amplified by PCR using different concentration in order to generate a standard curve (Figure 3A). The expression of Clo-miR-14 in plasma of RA and HC were also quantified (Figure 3B) after normalised with U6 loading control (Figure 3C) using a standard curve generated from commercial Clo-miR-14 mimic (Figure 3D). Clo-miR-14 was quantified in the plasma samples as well as in the turmeric rhizome, shown in tabular form (Table 1). The average concentration of Clo-miR-14 in RA (3.56 ng/200ng) was found to be 0.87-fold less compared with HC (4.00 ng/200 ng), and the difference was found to be non-significant (*P*≥0.05) (Figure 3E). The concentration of Clo-miR-14 was found to be much higher (≈29-fold), approximately 104 ng/200 ng of total isolated miRNA from turmeric compared with plasma samples (Table 1)

**Table 1:** Table showing the concentration of total miRNA and Clo-miR-14 miRNA in different plasma samples and *Curcuma longa* rhizome

S.No	Sample	miRNA concentration (ng/200 µl supernantant)	Clo-miR-14 concentration (ng/200 ng total miRNA)
1	RA 1	13.8	3.545
2	RA 2	39.3	3.499
3	RA 3	41.8	3.653
4	RA 4	6.9	3.663
5	RA 5	23.5	3.62
6	RA 6	18.9	3.55
7	RA 7	28.1	3.34
8	RA 8	16.3	3.52
9	HC 1	8.9	4.581
10	HC 2	17.8	3.91
11	HC 3	23.9	3.50
12	HC 4	28.8	3.871
13	HC 5	36,2	3.76
14	HC 6	25.1	3.92
15	HC 7	28.4	3.58
16	HC 8	30.1	3.78
17	*Curcuma longa*	132.7	104.219

### Clo-miR-14 regulates pro-inflammatory cytokines via nuclear factor-κB (NF-kB) pathway

**Figure 4:**
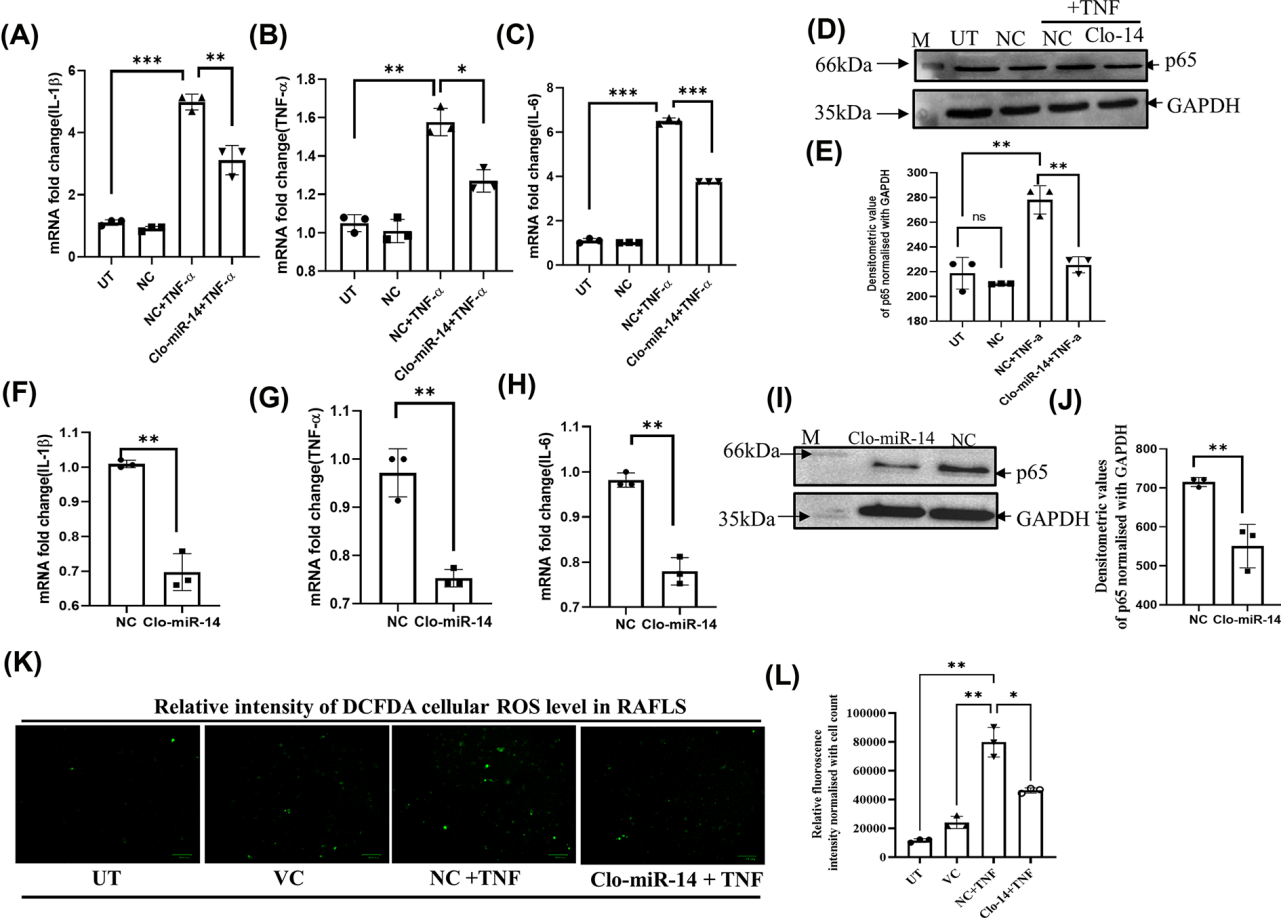
Anti-inflammatory and anti-rheumatic effect of Clo-miR-14 in SW982 and RA primary cells (**A**) The bar graph represents the fold change of IL-1β mRNA in the Clo-miR-14 treated group compared with the TNF-α treated group and controls in the SW982 cells (**B**) TNF-α mRNA level in Clo-miR-14 treated group compared with other groups. (**C**) IL-6 in the Clo-miR-14 treated group compared with other groups. (**D**) The image shows the Western Blot image of P65 (upper panel) and GAPDH (lower panel) as loading control of the Clo-miR-14 treated group compared with TNF-α treated group in SW982 cells. (**E**) The bar graph represents the densitometric value of the P65 level normalized with GAPDH in Western Blot in different treated groups. (**F**) The bar graph represents the mRNA fold change of IL-1β in the Clo-miR-14 treated group compared with the NC in primary RAFLS. (**G**) The mRNA level of TNF-α in Clo-miR-14 treated compared with NC in RAFLS. (**H**) The mRNA level of IL-6 in Clo-miR-14 treated compared with NC in RAFLS. (**I**) The Western blot image of P65 (upper panel) and GAPDH (lower panel) loading control of Clo-miR-14 treated group compared with NC. (**J**) The bar graph represents the densitometric value of the P65 level in primary RAFLS normalized with GAPDH loading control in Western blot. (**K**) The image shows the relative intensity of the DCFDA cellular ROS in RAFLS in different UT, VC, NC+TNF, and Clo-14+TNF groups. (**L**) The cellular ROS level was analyzed as relative intensity and represented by a bar graph which indicated a significant reduction in ROS level after treatment with Clo-miR-14 in RAFLS cells. DCFDA- 2′,7′, dichlorofluorescin diacetate; GAPDH, glyceraldehyde 3-phosphate dehydrogenase; HC, healthy control; IL, interleukin; M, Molecular weight marker; NC, non-specific miRNA pool control; RAFLS, rheumatoid arthritis fibroblast like synoviocyte; ROS, reactive oxygen species; TNF-α, tumor necrosis factor-α; UT, Untreated; VC, vehicle control; *P≤0.05, **P≤0.01, ***P≤0.001.

The mRNA analysis of pro-inflammatory cytokines (IL-1β, TNF-α, and IL-6) was carried out using RT-qPCR after Clo-miR-14 treatment in TNF-induced inflamed SW982 cells as well as in RAFLS. The mRNA analysis shows downregulation of IL-1β, TNF-α, and IL-6 by 0.6-, 0.8-, and 0.56-fold in SW982 cells (Figure 4A–C) and 0.68-, 0.77-, and 0.79-fold in RAFLS (Figure 4F–H) after Clo-miR-14 treatment in TNF-induced inflammation cells. Further, since NF-kB is the most common and prominent inflammatory pathway in RA, the NF-kB (P-65) level was also checked by Western blot (WB) in TNF-induced SW982 cells (Figure 4D) and primary RAFLS (Figure 4I). Upon analysis, we found that transfection of Clo-miR-14 results in the down-regulation of P-65 with 0.76-fold in SW982 cells (Figure 4E) and with 0.76-fold in RAFLS (Figure 4J). Our results thus confirmed that Clo-miR-14 plays a significant anti rheumatic and anti-inflammatory role in human via regulation of NF-kB pathway, as NF-kB is the major pathway affected in RA along with IL-1β, IL-6, and TNF-α pro-inflammatory cytokines.

### Clo-miR-14 ameliorates total reactive oxygen species production

The production of reactive oxygen species (ROS) leading to increased oxidative stress was analyzed by DCFDA assay after normalising with cell count (Supplementary Figure S2). The obtained fluorescence signal showed increased intracellular ROS production in TNF-α induced cells (NC+TNF) compared with untreated (UT) cells (Figure 4K). The ROS was significantly reduced by Clo-miR-14, which depicts its role in ROS scavenging activity (Figure 4L). The ROS reduction is one of the important parameters to check the inhibition of the NFkB pathway, considered as a major pathway affected in RA inflammation.

### Clo-miR-14 reduces the macroscopic joint score and pro-inflammatory cytokines in the CIA rat model

**Figure 5:**
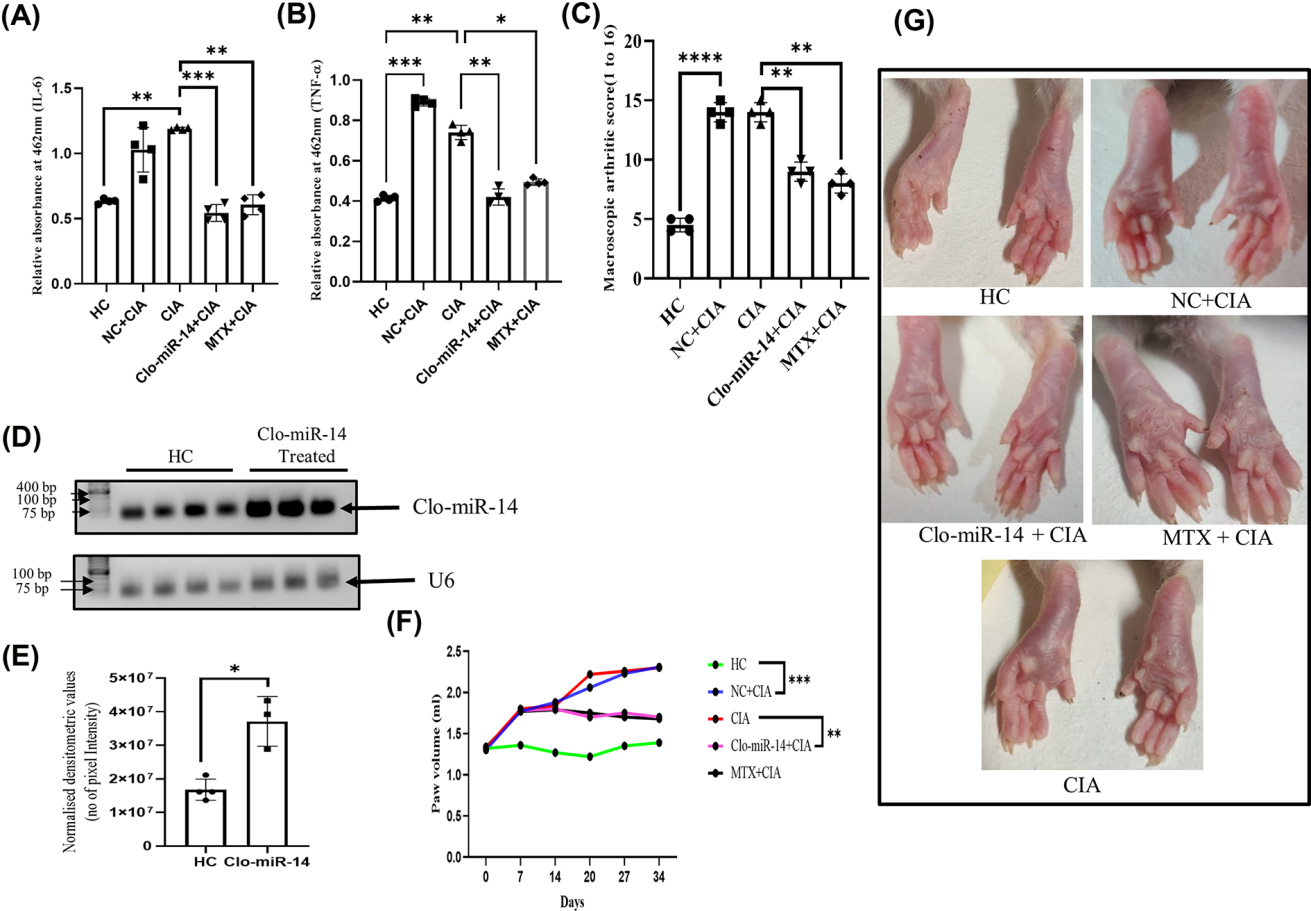
The effect of Clo-miR-14 on collagen-induced arthritis rat model (**A**) The bar graph represents the comparative optical density of IL-6 in rat plasma in HC (*N*=4), NC (*N*=4), CIA (*N*=4), Clo-miR-14 (*N*=4), and MTX (*N*=4) rat groups, which shows the downregulation of IL-6 level after Clo-miR-14 treatment compared with the CIA and NC. (**B**) The bar graph represents the optical density of TNF-α plasma level in rats, shown downregulated in Clo-miR-14 treated group plasma samples compared with the CIA and NC group. (**C**) The macroscopic arthritic score was measured on the 28th day and represented by a graph based on naked eye observation and swelling (based on measured paw volume by plethysmometer) in the Clo-miR-14 treated group compared with the CIA and NC groups. (**D**) The image showing the comparative level of Clo-miR-14 miRNA (upper panel) normalized with U6 loading control (lower panel) in rat synovium of HC (*N*=4) and Clo-miR-14 induced group (*N*=3). (**E**) The graph represents the densitometric value of Clo-miR-14 PCR product of Clo-miR-14 treated and HC rat synovium, run in 1.5% agarose gel normalized with U6 loading control which depicts the level of Clo-niR-14 in rat synovium. (**F**) The paw volume was measured using a plethysmometer from day 0 to day 34, and graphs were plotted to find the changes in paw volume in the Clo-miR-14 treated group compared with the CIA, VC, and MTX groups. (**G**) Representative hind paw image of the rats from each group, where edema and redness were seen reduced in the Clo-miR-14 and MTX (standard drug) treated group compared with the CIA and NC groups. CIA, collagen-induced arthritis; HC, healthy control; IL, interleukin; M, molecular weight marker; MTX, methotrexate; NC, non-specific miRNA pool control; TNF-α, tumor necrosis factor-α; VC, vehicle control; **P≤0.01, ***P≤0.001, ****P≤0.0001.

To mimic the* in vivo* RA condition, the CIA rat model was generated by collagen induction followed by intraperitoneal (IP) Clo-miR-14 treatment. Rat plasma samples were collected after scarification from all the rats and were used to measure cytokines. Our results suggested that Clo-miR-14 significantly reduced IL-6 (Figure 5A) and TNF-α levels (Figure 5B) with 0.55- (*P* =0.0004) and 0.58- (*P*=0.0043) fold, respectively, after Clo-miR-14 treatment compared with CIA and non-specific miRNA control (NC) group. The macroscopic arthritic score was also found significantly (*P*=0.0051) reduced in Clo-miR-14 treated group compared with non-specific miRNA control and CIA groups (Figure 5C). Methotrexate (MTX) was used as a standard drug [32] and macroscopic arthritic score of MTX group was observed to have similar effect as of Clo-miR-14 treated CIA group (Figure 5C) indicating that Clo-miR-14 has potential in reducing effect on RA like inflammation. The delivery of Clo-miR-14 in the synovium was validated by measuring Clo-miR-14 level in rat synovium after isolation of total miRNA in Control and the Clo-miR-14 treated group by RT-PCR and by running the PCR product on agarose gel (Figure 5D), where it was observed elevated by 2.1-fold (*P*=0.013) compared with the HC (Figure 5E). After day 14, the Clo-miR-14 group and MTX group showed reduction in paw volume till 34th day (Figure 5F) compared with the CIA and NC group (Figure 5F). The results were supported by the hind paw images taken on day 34, the redness and swelling of paw were reduced by 0.64- and 0.57-fold, respectively, in the group treated with Clo-miR-14 and MTX compared with the CIA and NC groups (Figure 5G).

### Histological analysis confirms the anti-inflammatory effect of Clo-miR-14

**Figure 6:**
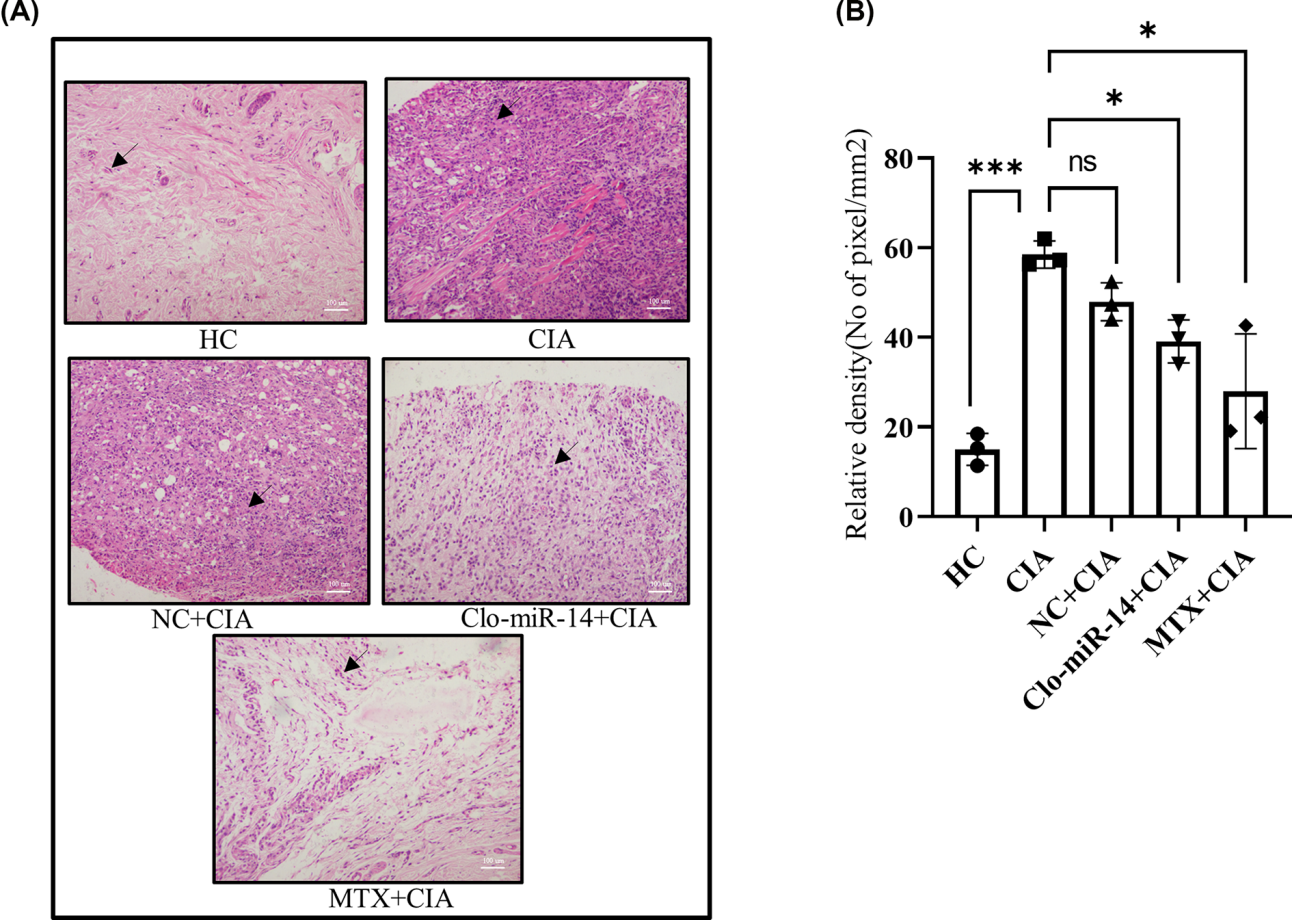
Histological analysis of inflammation in CIA rat synovium (**A**) The H&E staining shows decreased inflammation (pink color) in Clo-miR-14 treated (*N*=4) and in MTX (*N*=4) groups compared with NC (*N*=4) CIA (*N*=4), and HC (*N*=4) groups. The arrow depicts cells in the synovium (**B**) The graph represents the analysis of cell infiltration in the synovium of different rat groups, which was measured and found to be down-regulated in the Clo-miR-14 (*N*=3) treated group compared with the CIA (*N*=3), and NC (*N*=3) group. CIA, collagen-induced arthritis; H&E, Hematoxylin and Eosin; HC, healthy control; NC, non-specific miRNA pool control; MTX, Methotrexate; ns, *P≤0.05, ***P≤0.001.

The pink colour in Hematoxylin and Eosin staining (H&E) showed the level of inflammation in the synovium [30], and the purple colour indicates the number of nuclei present (marked with a black arrow) in the synovium, used to calculate the number of cells infiltrated into the given area (Figure 6A) due to inflammation [33]. The H&E images were analyzed by using ImageJ to calculate the relative density with normalised cell numbers. The analysis of mean densitometry value indicates that the inflammation was reduced in the Clo-miR-14 and MTX (positive control) treated groups by 0.66- and 0.45-fold, respectively, compared with the CIA and NC group (Figure 6B).

## Discussion

The mi-RNAs present in various medicinal plants are less explored in terms of RNA-based therapeutic [34]. The Report shows that all essential biological pathways, such as glucose metabolism, cellular differentiation, proliferation, apoptosis, and immune response, are regulated by miRNAs [35,36]. Many studies have reported that various medicinal plant products (fruits, leaves, stems, roots, etc.) possess anti-inflammatory and anti-rheumatic properties. However, consumption of such plant products has also been reported to cause side effects [37,38]. It is, therefore, essential to identify the exact component of medicinal plants to be beneficial as an anti-inflammatory component with no side effect.

Currently, miRNA drug therapy is utilized as the most recent and advance technique in various inflammatory disease such heart disease [39,40]. Since exogenous miRNA from plant origin triumphs their target and regulate gene expression after it’s absorption through diet, we focused our study to explore the potentiality of plant miRNAs present in orally consumed medicinal spices [4,35]. We randomly selected four Indian spices (turmeric, fenugreek, kalonji, and flax seed) that are regularly consumed, specifically by the Indian population. We observed a significant inhibition of well-known pro-inflammatory cytokines levels (IL-1β, IL-6, and TNF-α) in the *in vitro* model of RA by total miRNAs extracted from Turmeric and fenugreek (Figure 1A–C). This substantiates their anti-rheumatic and anti-inflammatory properties. The miRNA level of the other two medicinal plant products (kalonji and flax seed) was revealed to have anti-inflammatory, but they do not have anti-rheumatic potentiality.However turmeric and fenugreek show both anti-rheumatic and anti-inflammatory properties. Further analysis revealed that turmeric has a maximum reduction of TNF-α. Also, our previous report [2] suggests that turmeric consists Clo-miR-14 miRNA, which has potential anti-rheumatic and anti-inflammatory properties. However, the stability of Clo-miR-14 remains unclear. In order to fill this gap, we focused our study to specifically recognize the significance of miRNA activity in their absorption from processed foods and to assess the stability of miRNAs under cooking temperatures.

Earlier, MIR168a from rice has been reported to be stablein the plasma and was validated for their absorption in the diet [4]. But the stability of various miRNAs at cooking temperatures remains unexplored to the best of our knowledge. In our earlier studies, we identified ‘Clo-miR-14’ as one of the potential anti-rheumatic exogenous miRNA from turmeric found stable in FBS [2]. We explored further, in the present study, and confirmed that, Clo-miR-14 can bear 100°C (near cooking temp) for at least 50 min without any significant loss in their integrity (Figure 2C,D). The prime reason for this robust stability of plant miRNA may be because of the slower degradation of plant miRNA compared with mammalian miRNAs, and this may be due to the presence of 2'-O-methylation at 3'-ribose sugar which is playing protective role [4,5,7]. Further, higher concentration of Clo-miR-14 in the rhizome of turmeric compared with blood samples indicates that turmeric is one of the possible sources of Clo-miR-14 (Figure 3A) and is only possible if the Clo-miR-14 is stable enough to sustain in the human digestive system. Since there is a lack of miRNA database for fenugreek, kalonji, and flax seed, we could not confirm the presence of Clo-miR-14 in these samples. The concentration of miRNA in the blood also depends on the half-life of plant miRNA in blood, which ranges from ≈ 1.5 h to more than 13 h and may also affect Clo-miR-14 concentration after consumption [41]. Earlier, the absorption of plant miRNA in the human body and the validation of their role in human gene regulation have been reported [3]. Report suggests that Clo-miR-14 shares sequence similarity with human miRNA hsa-miR-4693-5p, known to target LGSN, TMEM200C, Short-stature homeobox 2 (SHOX2), and MAP3K9 gene [42]. The SHOX2 exhibited matrix metalloproteinase (MMP) activation, and collagen-II expression, involves in cartilage degradation and reduced joint health [43]. Cartilage degradation also exhibited increased proliferation in RAFLS, characteristics of RA [43]. Thus, taken together, we presumed that cartilage degradation is possibly regulated by Clo-miR-14 due to its sequence similarity with has-miR-4693 and its stability in human plasma, suggesting a possible contribution to the regulation of pathways that culminate in reduced inflammation. Further, to validate the functional role of Clo-miR-14 in attenuating RA conditions, synovial fibroblast cells SW982 and RAFLS were treated with synthetic Clo-miR-14 with modified 2′-O-methylation [18]. We used RAFLS, since it provides the actual disease condition for testing in human subjects and the insight biological consequence during the disease conditions [44]. Our results confirmed that Clo-miR-14 has an anti-rheumatic and anti-inflammatory effect in the *in vitro* models of RA using SW982 (Figure 4A–C) as well as in RAFLS (Figure 4F–H). Further, the ant-inflammatory effect was validated by Nf-kB p65 protein level in SW982 and in RAFLS (Figure 4E,J). The NF-κB signaling pathway, stimulated by IL-1β, promotes inflammatory responses in chondrocytes [45] elevating the expression of pro-inflammatory cytokines genes [46]. The NF-κB signaling pathway is therefore generally considered to be the potential therapeutic target in the progression of inflammatory disease like RA [47,48]. Our study suggests that Clo-miR-14 has a potential in regulating pro-inflammatory cytokines IL-6, TNF-α (Figure 5A,B), macroscopic arthritic score in CIA rat model (Figure 5C), and infiltration of immune cells, a hallmark of RA symptoms on histological evidence [49]. All the above parameters were found to be reduced significantly in the Clo-miR-14 treated group compared with the CIA group (shown by black arrow Figure 6A,B). Further, it is known that RA is accomplished with the production of ROS in the synovium, which we have found inhibited by Clo-miR-14 induction (Figure 4K,L). This may be due to the result of TNF reduction, since TNF has been suggested as one of the potential targets for Clo-miR-14 [2].

## Conclusion

Our results, therefore, suggest that, Clo-miR-14, abundantly present in Turmeric, has potential anti-inflammatory and anti-rheumatic effects, and also stable in processed food, resulting in significant amelioration of pro-inflammatory cytokines and arthritic score, can be considered as a potential miRNA-based drug candidate for RA. However, the optimum concentration must be determined along with bioavailability for every individual miRNA, before concluding its efficacy in treating the disease. Our study also has some limitation since many more Indian spices have medicinal properties, could not be included in the present study, and can be explored in future studies.

## Supplementary Material

Supplementary Figures S1-S4

## Data Availability

All the data and raw files were submitted to the corresponding author for all original figure and detail protocol. Please contact Dr Sagarika Biswas (Mail: sagarika.biswas@igib.res.in) for more detail.

## Abbreviations

CIA: collagen-induced arthritis
H&E: Hematoxylin and Eosin
IL: interleukin
MMP: matrix metalloproteinase
RA: rheumatoid arthritis
RAFLS: rheumatoid arthritis fibroblast-like synovial cells
SHOX2: short-stature homeobox 2
TNF-α: tumor necrosis factor-α
