# Supplementary material for: Clo-miR-14: a medicinally valued spice-derived miRNA with therapeutic implications in rheumatoid arthritis
Source: Biosci Rep. 2024 Sep 10;44(9):BSR20240311. doi: 10.1042/BSR20240311 (PMC11392912; doi:10.1042/BSR20240311)
Supplement: Supplementary Figures S1-S4 [file BSR-2024-0311_supp.pdf]

Supplementary data

Figure S1

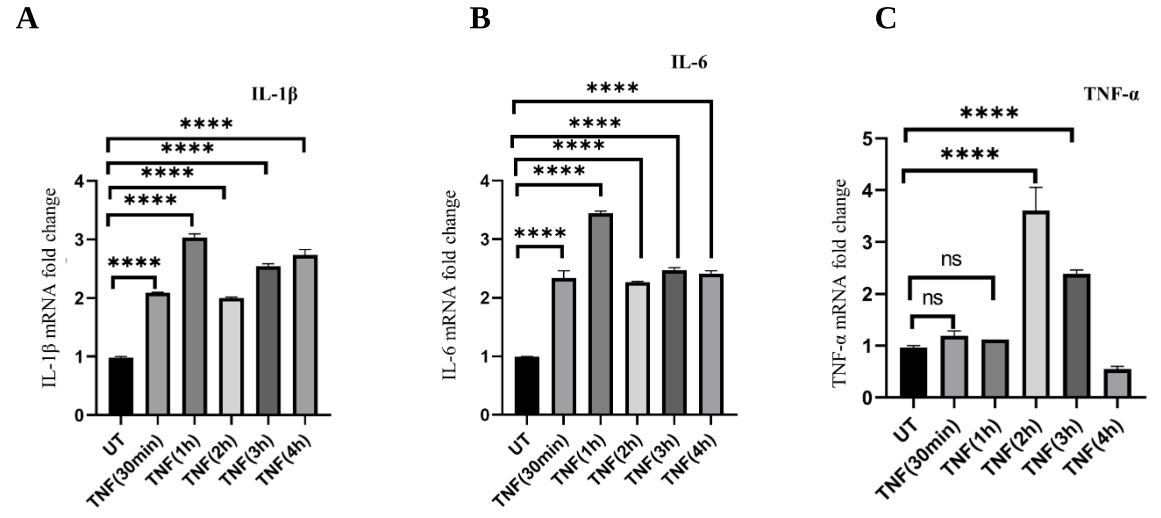

Supplementary Figure S1: The standardization of TNF-α dose to induce inflammation in SW982 cells (A) The image shows IL-1β mRNA expression after induction of TNF-α at different time points (30 min, 1h, 2h, 3h, and 4h). (B) The image shows IL-16 mRNA expression after induction of TNF-α at different time points (30 min, 1h, 2h, 3h, and 4h). (C) The image shows TNF-α mRNA expression after induction of TNF-α at different time points (30 min, 1h, 2h, 3h, and 4h).

Figure S2

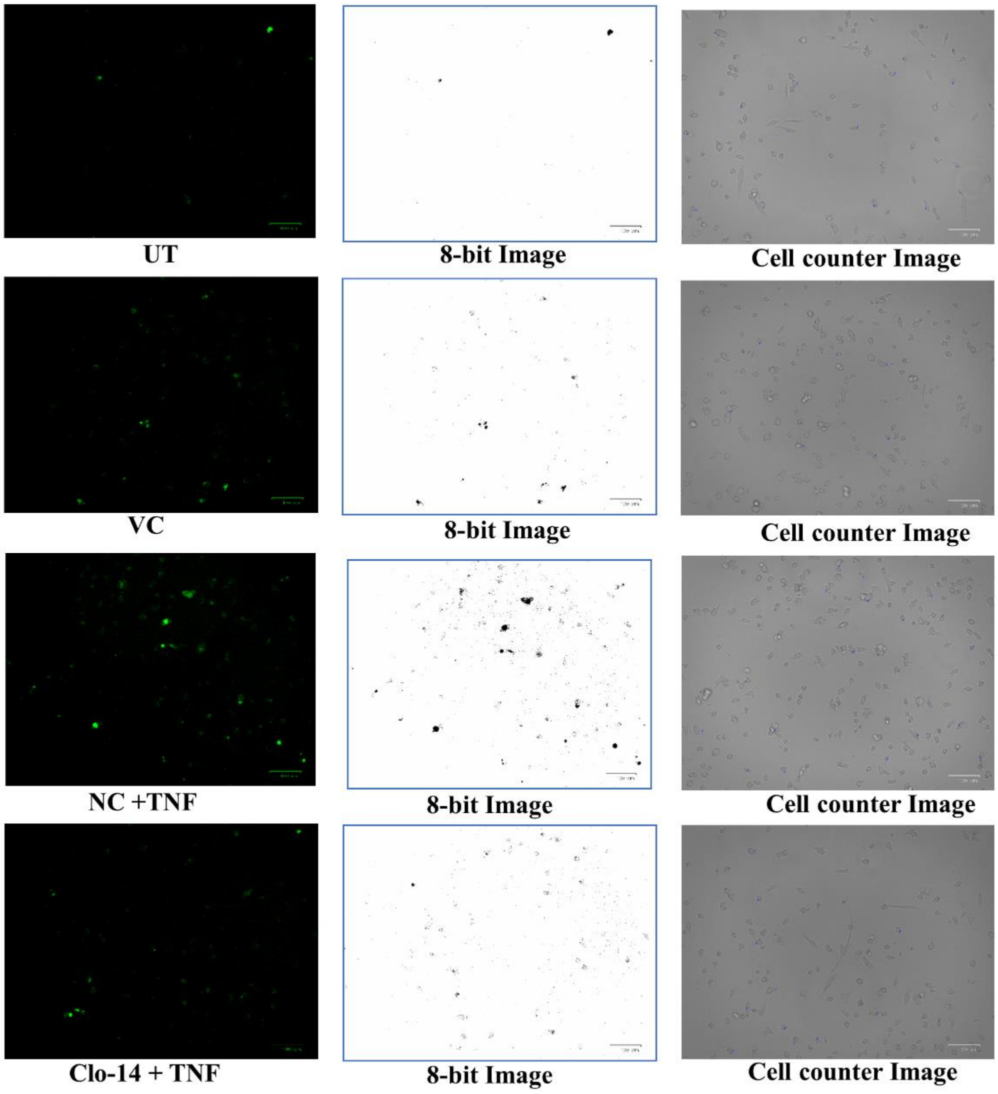

Supplementary figure S2: The image showing DCFDA cellular ROS level in UT, VC, NC+TNF, and Clo-14+TNF groups as a function of fluorescence in the RAFLS cells. The respective 8 bit image also shown next to the fluorescence image respectively followed by their normalized bright field image for counting the cells. The fluorescence was normalized with the cell count with the help of the bright field image.

Figure S3

RAW image for represented Figure 4D

A **B**

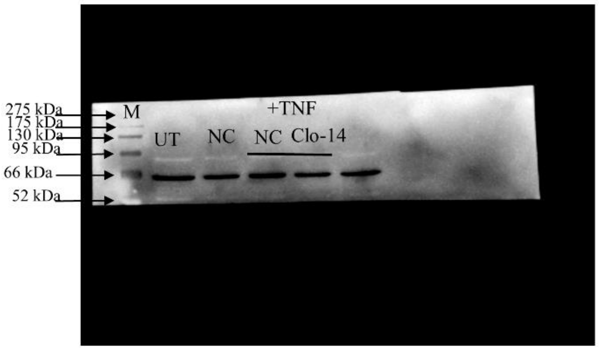

Upper panel(p65)

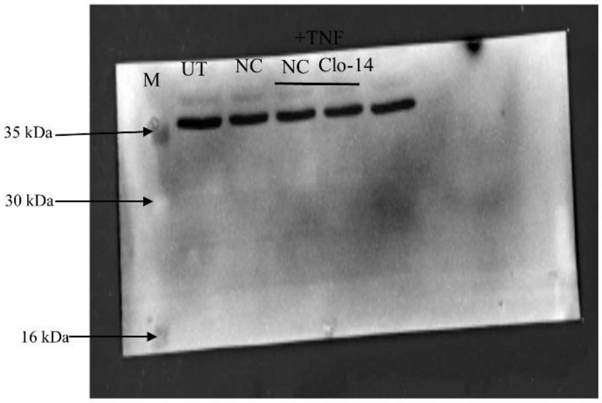

Lower panel(GAPDH)

Figure S3: The image showing raw western-blot image of p-65(A), and GAPDH loading control (B) in SW982 cell line after treatment.

Figure S4

RAW image for represented Figure 4I

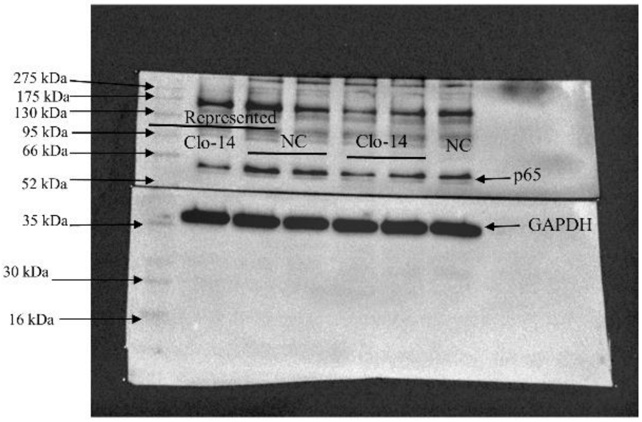

Figure S4: The image showing western-blot image of p65 (upper panel), and GAPDH loading control (lower panel) in RAFLS primary cells.
